# Infectious complications following heart transplantation in the era of high-priority allocation and extracorporeal membrane oxygenation

**DOI:** 10.1186/s13613-019-0490-2

**Published:** 2019-01-25

**Authors:** Stéphanie Pons, Romain Sonneville, Lila Bouadma, Lenka Styfalova, Stéphane Ruckly, Mathilde Neuville, Aguila Radjou, Jordane Lebut, Marie-Pierre Dilly, Bruno Mourvillier, Richard Dorent, Patrick Nataf, Michel Wolff, Jean-François Timsit

**Affiliations:** 1 Medical and Infectious Diseases Intensive Care Unit, AP-HP, Bichat-Claude Bernard University Hospital, 46 rue Henri Huchard, 75877 Paris Cedex, France; 2 UMR 1148, LVTS, Sorbonne Paris Cité, Inserm/Paris Diderot University, Paris, France; 3 UMR 1137, IAME Team 5, DeSCID: Decision Sciences in Infectious Diseases Prevention, Control and Care, Sorbonne Paris Cité, Inserm/Paris Diderot University, Paris, France; 4 Department of Biostatistics, ICUREsearch, Paris, France; 5 Department of Anesthesiology, AP-HP, Bichat-Claude Bernard University Hospital, Paris, France; 6 Department of Cardiac Surgery, AP-HP, Bichat-Claude Bernard University Hospital, Paris, France

**Keywords:** Heart transplantation, Infections, Extracorporeal membrane oxygenation, Outcome

## Abstract

**Background:**

Infectious complications are a major cause of morbidity and mortality after heart transplantation (HT). However, the epidemiology and outcomes of these infections in the recent population of adult heart transplant recipients have not been investigated.

**Methods:**

We conducted a single-center retrospective study on infectious complications occurring within 180 days following HT on consecutive heart transplant recipients, from January 2011 to June 2015 at Bichat University Hospital in Paris, France. Risk factors for non-viral infections occurring within 8, 30 and 180 days after HT were investigated using competing risk analysis.

**Results:**

Overall, 113 patients were included. Fifty-eight (51%) HTs were high-priority allocations. Twenty-eight (25%) patients had an extracorporeal membrane oxygenation (ECMO) support at the time of transplantation. Ninety-two (81%) patients developed at least one infection within 180 days after HT. Bacterial and fungal infections (*n* = 181 episodes) occurred in 80 (71%) patients. The most common bacterial and fungal infections were pneumonia (*n* = 95/181 episodes, 52%), followed by skin and soft tissue infections (*n* = 26/181, 14%). Multi-drug-resistant bacteria were responsible for infections in 21 (19%) patients. Viral infections were diagnosed in 44 (34%) patients, mostly Cytomegalovirus infection (*n* = 39, 34%). In multivariate subdistribution hazard model, prior cardiac surgery (subdistribution hazard ratio sHR = 2.7 [95% CI 1.5–4.6] *p* < 0.01) and epinephrine or norepinephrine at the time of HT (sHR = 2.3 [95% CI 1.1–5.2] *p*  = 0.04) were significantly associated with non-viral infections within 8 days after HT. Prior cardiac surgery (sHR = 2.5 [95% CI 1.4–4.4] *p* < 0.01), recipient age over 60 years (sHR = 2.0 [95% CI 1.2–3.3] *p* < 0.01) and ECMO following HT (sHR = 1.7 [95% CI 1.0–2.8] *p* = 0.04) were significantly associated with non-viral infection within 30 days after HT, as well as within 180 days after HT.

**Conclusion:**

This study confirmed the high rate of infections following HT. Recipient age, prior cardiac surgery and ECMO following HT were independent risk factors for early and late bacterial and fungal infections.

**Electronic supplementary material:**

The online version of this article (10.1186/s13613-019-0490-2) contains supplementary material, which is available to authorized users.

## Introduction

Heart transplantation (HT) is the gold standard treatment of end-stage cardiac failure [1]. Substantial changes have recently occurred in the epidemiology of HT, including older patients and more high-priority allocations for critically ill patient [2–4]. Moreover, ventricular assist device (VAD) as a bridge to HT and extracorporeal membrane oxygenation (ECMO) support before and after HT has significantly increased in the last years [5]. Despite a high incidence of infections in these immunocompromised patients, recent data describing their epidemiology following HT are scarce. Most studies in adult heart transplant recipients conducted years ago reported an incidence of bacterial infections of 20–30% [6–11]. More recently, studies have found a 9–45% Cytomegalovirus (CMV) infection rate [7, 11–14]. Infections in heart transplant recipients represent a real challenge, as they are responsible for increased morbidity, mortality, length of stay and associated costs [6, 7]. Moreover, many changes in patient care have occurred in the last decade, including new immunosuppressive protocols, advances in early infection diagnosis and multi-drug-resistant pathogens emergence. Understanding the current epidemiology of infections and their clinical impact on outcome may help to better prevent and treat patients in the future.

The purpose of this study was to describe the epidemiology and outcomes of infections occurring within 180 days after HT. Specifically, we aimed to determine risk factors for non-viral infections occurring within 8, 30 and 180 days following HT.

## Materials and methods

### Patient population and study design

This single-center retrospective study was conducted at Bichat-Claude Bernard Hospital, Paris, France. We included all consecutive adult heart transplant recipients between January 1, 2011, and June 30, 2015. Data on donors and recipients were retrieved from the medical records and the French Transplant Agency database “Cristal,” which is a prospectively maintained database designed to store all the donors and recipients’ data. The French Intensive Care Society Ethics Committee approved the study (CE SRLF17-10). Data on intensive care unit (ICU) management and infections were retrospectively collected. In accordance with French law, all the patients or next of kin were informed of the possibility to use their hospitalization’s data for clinical research and gave their consent.

### Immunosuppressive and antimicrobial prophylaxis after transplantation

Immunosuppressive treatment and antimicrobial prophylaxis for heart transplant recipient are well codified in our transplantation center, in accordance with the International Society of Heart and Lung Transplantation (ISHLT) guidelines [15]. All patients received intraoperative methylprednisolone and mycophenolate mofetil intravenous boli, and an early postoperative induction therapy with either antithymocyte globulins or basiliximab. Then, a triple maintenance therapy with calcineurin inhibitors, mycophenolate mofetil and steroids was administered. For highly sensitized patients, plasma exchanges before and/or after HT were realized.

Intraoperative antibiotic prophylaxis was cefazolin except otherwise decided (ongoing infection or multi-drug-resistant (MDR) bacterium colonization). The patients were systematically screened for methicillin-resistant *Staphylococcus aureus* or extended spectrum beta lactamase (ESBL) producing *Enterobacteriaceae* carriage before HT. Postoperative prophylaxis against infections was initiated between day 3 and day 7 after heart transplantation. Postoperative cotrimoxazole was administered for *Pneumocystis jiroveci* pneumonia and toxoplasmosis prevention for a duration varying from 1 year to lifelong, depending on the risk (i.e., immunosuppressive regimen, toxoplasmosis mismatch). Oral amphotericin B was given to all patients within 30 days after HT in the absence of intestinal disease. CMV disease prevention consisted in antiviral prophylaxis with valganciclovir for high-risk patients (donor positive/recipient negative for CMV). For CMV seropositive patients or CMV seronegative donors and recipients, the preemptive strategy based on the level of plasmatic CMV copies was chosen (threshold 4 log^10^ copies/mL). In case of treated acute rejection, valganciclovir prophylaxis was systematically administered in CMV seropositive recipients, associated with cotrimoxazole prophylaxis in case of interruption. Heart transplant recipients received, if possible, influenza and pneumococcal vaccines before heart transplantation, or within the first year following the transplantation, as recommended by our local protocol.

### Clinical definitions

Infections were defined as per the CDC guidelines [16]. The definitions of infectious complications in heart transplant recipients also followed the 2010 ISHLT work and the American Society of Transplantation recommendations [17, 18]. Only microbiologically documented infections were included in the analysis. MDR bacteria included lack of susceptibility to one or more agents in three or more antimicrobial categories active against the isolated bacteria [19]. CMV infection was defined as CMV virus detected by viral culture or quantitative PCR assay for CMV in any body fluid or tissue specimen. Primary CMV infection was defined as the first detection of CMV infection in an individual who had no evidence of CMV exposure before transplantation and was classified as CMV infection. CMV disease was defined as the presence of appropriate clinical symptoms and/or signs together with documentation of CMV in tissue from the relevant organ [20]. Diagnosis of proven or probable invasive fungal infection was based on histopathological findings or blood/tissue culture. All the infectious complications occurring within 180 days after HT were recorded. Primary and secondary graft dysfunctions were defined according to the ISHLT consensus conference on primary graft dysfunction after cardiac transplantation [21]. Thus, failure to wean from the extracorporeal circulation at the end of the surgery or need of a circulatory support by ECMO within 24 h after HT defined a severe graft dysfunction. Acute rejection was defined according to the original ISHLT Heart Biopsy Grading Scale [22].

### Statistical analysis

Quantitative parameters are reported as median and interquartile range [IQR 25–75 percentile] and qualitative parameters as number and percentage. Univariate and multivariate analyses were performed to assess for associations between potential risk factors and non-viral infections within 8, 30 and 180 days after HT using a Fine and Gray subdistribution hazard model considering death as a competing risk. Factors included in the multivariate model were selected among variables yielding *p* values smaller than 0.10 in univariate analysis and adjusted on the sequential organ failure assessment at the time of HT. Subdistribution hazard ratio (sHR) and 95% confidence interval (95% CIs) were estimated for significant risk factors, based on a multivariate analysis. The impact of infectious complications treated as time-dependent events on mortality at 90 days after HT was determined using Cox regression models. Statistics were performed using SAS 9.4 and R softwares, and a *p* value of 0.05 or lower was considered significant.

## Results

### Patients

Overall, 113 patients underwent HT between January 1, 2011, and June 30, 2015, at Bichat-Claude Bernard Hospital, Paris. All those patients were included in the study, with no lost to follow-up within 180 days after HT. Patient’s baseline characteristics are given in Table 1. Fifty-eight (51%) HTs were national high-priority allocations. Patients were predominantly males (*n* = 86, 76%), with a median age of 53 [40; 62] years. Main causes of cardiac failure were dilated cardiomyopathy (*n* = 50, 44%) and ischemic cardiomyopathy (*n* = 44, 39%). Most patients (*n* = 79, 70%) were already hospitalized at the time of HT, including a large number in the ICU (*n* = 44, 39%). Twenty (18%) patients had VAD as a bridge to transplantation, and 28 (25%) patients were under ECMO support at the time of HT.

**Table 1:** Heart transplant recipient baseline characteristics

Characteristics	Heart transplant recipients (*n* = 113)
Recipient gender, male; *n* (%)	86 (76)
Recipient age, years; median [IQR]	53 [40; 62]
Prior diabetes mellitus; *n* (%)	24 (21)
Prior cardiac surgery (VAD surgery excluded); *n* (%)	27 (24)
BMI, kg/m^2^; median [IQR]	24,4 [22, 1; 27, 8]
Serum creatinine (μmol/l); median [IQR]	130 [99; 158]
Etiology of heart failure; *n* (%):	
Dilated cardiomyopathy	50 (44)
Ischemic cardiomyopathy	44 (39)
Hypertrophic cardiomyopathy	8 (7)
Others	11 (10)
Recipient location right before HT; *n* (%)	
Home	34 (30)
Medical/surgical units	14 (12)
Intermediate care units	21 (19)
Intensive care units	44 (39)
Length of stay before HT, days; median [IQR]	
Hospital	11 [6; 19]
Intermediate or intensive care units	8 [5; 14]
Donor age, years; median [IQR]	45 [37; 42]
Donor gender, male; *n* (%)	67 (59)
CMV mismatch (D+/R−); *n* (%)	15 (13)
VAD before HT; *n* (%)	20 (18)
ECMO before HT; *n* (%)	28 (25)
SOFA score at time of HT; median [IQR]	4 [1; 6]
National high-priority allocation; *n* (%)	58 (51)
Cold ischemia time, minutes; median [IQR]	190 [140; 210]
Plasma exchanges after HT; *n* (%)	20 (18)

*BMI* body mass index, *CMV* cytomegalovirus, *D* donor, *ECMO* extracorporeal membrane oxygenation, *HT* heart transplantation, *R* recipient, *SOFA score* sequential organ failure assessment score, *VAD* ventricular assist device

### Epidemiology and etiologies of infectious complications

**Fig. 1:**
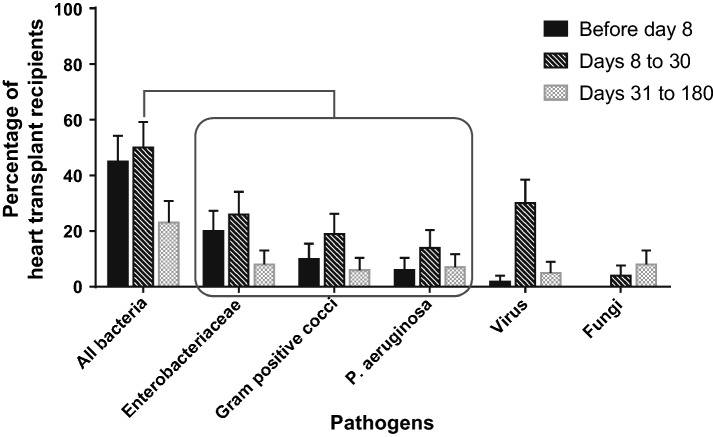
Percentage of adult heart transplant recipients (*n* = 113) infected by different pathogens according to the postoperative period (before day 8, days 8 to 30 and days 31 to 180*). *A same patient can have been infected multiple times by the same pathogen and during different time periods

Twenty (18%) patients of our cohort, including six VAD patients, had an infection within 8 days before heart transplantation. However, device infection was the main reason for bridging them to transplantation in only two cases. Twenty-two (19%) patients received an antibiotic therapy for an ongoing infection or an alternative prophylaxis other than cefazolin during heart transplantation, preferentially targeting ESBL (*n* = 6), Staphylococci (*n* = 6), *Pseudomonas aeruginosa* (*n* = 3), other Gram-negative bacilli (*n* = 4) and other pathogens (*n* = 3). Ninety-two (81%) patients developed at least one infection within 180 days after HT. There were 181 episodes of bacterial and fungal infections occurring in 80 (71%) patients. Fifty-five (49%) patients had at least one bacterial infection within 8 days after HT. Although bacteria were the most common identified pathogens in the early postoperative phase, viruses also accounted for a high proportion of infections between 8 and 30 days following HT (Fig. 1). Data on infections and outcomes in heart transplant recipients within 180 days after heart transplantation are described in Table 2. The most common bacterial and fungal infections were pneumonia (*n* = 95/181 infectious episodes, 52%), followed by skin and soft tissue infections (*n* = 26, 14%). We recorded 30 bloodstream infections (BSI) occurring after a median time of 10 [5–21] days following heart transplantation. Of these BSIs, 12/30 (40%) were secondary to another infection (pneumonia *n* = 4, catheter-related infection *n* = 3, cannulas-related infection *n* = 3, intra-abdominal infection *n* = 2) and 18/30 (60%) were of unknown origin. Six out of eighteen patients with BSI of unknown origin were under ECMO.

**Table 2:** Infectious complications and outcomes in heart transplant recipients within 180 days after heart transplantation

Events	Overall population (*n* = 113)
Deaths; *n* (%)	19 (17)
Patients with ≥ 1 post-transplant infection(s) of all pathogens; *n* (%)	92 (81)
Number of post-transplant non-viral infectious episodes	181
Site of non-viral infections; *n* (% infectious episodes)^a^	
Respiratory tract	95 (52)
Skin and soft tissues	26 (14)
Urinary tract	22 (12)
Bloodstream infection of unknown origin	18 (10)
Secondary bloodstream infections	12 (7)
Mediastinitis	11 (6)
Catheter-related infection	8 (4)
Other^b^	13 (7)
Patients with at least an infection caused by; *n* (%)^c^	
Overall bacteria and fungi	80 (71)
Gram-negative bacilli	63 (56)
*Enterobacteriaceae*	50 (44)
*P. aeruginosa*	24 (21)
Gram-positive cocci	35 (31)
*S. aureus*	4 (3)
Enterococci	19 (17)
Streptococci	10 (9)
Multi-drug-resistant bacteria	21 (19)
Fungi	16 (14)
*Candida* spp.	4 (4)
*Aspergillus* spp.	6 (5)
Other^d^	6 (5)
Patients with a viral infection; *n* (%)	44 (39)
CMV infection	39 (34)
HSV infection	10 (9)
Patients with treated acute rejection; *n* (%)	24 (21)

Multi-drug-resistant bacteria: lack of susceptibility to one or more agents in three or more antimicrobial categories active against the isolated bacteria

*CMV* cytomegalovirus, *HSV* herpes simplex virus

^a^One episode could be counted multiple times according to the number of infectious sites

^b^Other infections: 8 gastrointestinal tract infections, 2 central nervous system infections and 3 other infections

^c^A same patient could have been infected by different germs during the same period

^d^Other invasive fungal infections: (*Fusarium* spp. *n* = 1, *Pneumocystis jiroveci n* = 1, *Microsporidium* spp. *n* = 1, *Trichosporon* spp. *n* = 1, undefined yeast *n* = 2)

Within 180 days after HT, 63 (56%) patients developed at least one infection caused by Gram-negative bacilli. *Enterobacteriaceae* were the most common bacteria responsible for infections within 8 days after HT (22 patients, 20%). Bacterial infections due to *Pseudomonas aeruginosa* preferentially occurred after 8 days following HT. Thirty-five (31%) patients developed at least one infection due to Gram-positive cocci, mostly *Enterococcus* spp. (19 patients, 17%). Twenty-two (19%) patients were colonized by a MDR bacterium before transplantation, and 6 (5%) acquired one during the first 8 days in the ICU. Nine (8%) colonized patients developed at least one infection due to the same MDR microorganism during their ICU stay. Overall, MDR bacteria were responsible for infections in 21 (19%) patients, mostly ESBL (*n* = 14, 12%). Sixteen patients presented at least one invasive fungal infection. *Aspergillus* spp. were responsible for invasive infection in 6 (5%) patients, in all cases over 30 days following HT. *Candida* spp. were considered responsible for infection in 4 (4%) patients. Viral infections were diagnosed in 44 (39%) patients, mostly CMV infection (*n* = 39, 34%), occurring preferentially between 8 and 30 days after HT (Fig. 1). All CMV infections were asymptomatic and treated preemptively, according to the level of plasmatic CMV copies.

### Risk factors for postoperative non-viral infections

The pre-, intra- and postoperative patients’ characteristics with at least one bacterial or fungal infection or without any non-viral infection within 8, 30 and 180 days after HT were firstly compared by univariate analysis (Additional file 1: Table S1). All non-viral infections diagnosed within 8 days following HT were bacterial infections. There was no significant difference in the induction and maintenance immunosuppressive regimen between groups. In multivariate analysis, prior cardiac surgery (VAD excluded) (sHR = 2.7 [95% CI 1.5–4.6] *p* < 0.01) and epinephrine or norepinephrine continuous infusion at the time of HT (sHR = 2.3 [95% CI 1.1–5.2] *p* = 0.04) were significantly associated with the occurrence of bacterial infection within 8 days after HT. Within 30 days after HT, prior cardiac surgery (VAD excluded) (sHR = 2.5 [95% CI 1.4–4.4] *p* < 0.01), recipient age over 60 years (sHR = 2.0 [95% CI 1.2–3.3] *p* < 0.01) and ECMO within 24 h after HT (sHR = 1.7 [95% CI 1.0–2.8] *p* = 0.04) were significantly associated with bacterial and fungal infections. Lastly, within 180 days after HT, prior cardiac surgery (sHR = 2.3 [95% CI 1.3–4.0] *p* < 0.01), recipient age over 60 years (sHR = 1.8 [95% CI 1.1–3.1] *p* = 0.02) and ECMO within 24 h after HT (sHR = 1.6 [95% CI 1.0–2.7]) *p* = 0.05) were significantly associated with non-viral infections (Table 3).

**Table 3:**
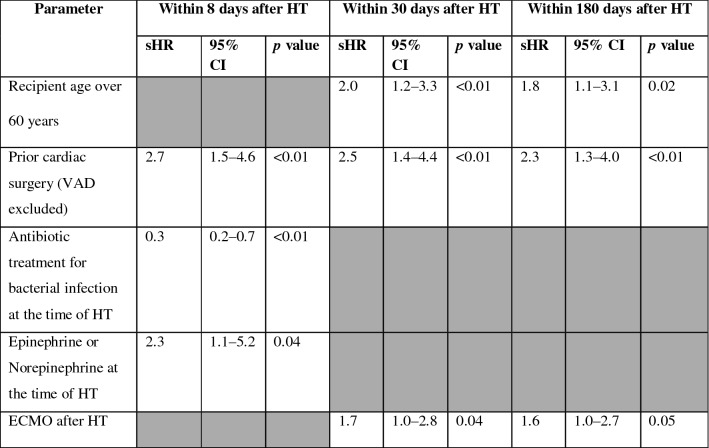
Multivariate Fine and Gray analysis of risk factors associated with the occurrence of bacterial and fungal infections within 8 days, 30 days and 180 days after heart transplantation adjusted on SOFA score at the time of heart transplantation

*ECMO* extracorporeal membrane oxygenation, *HT* heart transplantation, *SOFA score* sequential organ failure assessment score, *VAD* ventricular assist device

*Only significant variables with *p*-value < 0.1 in the univariate model and SOFA score at time of heart transplantation were included in the multivariate model

### Incidence of early severe graft dysfunctions

Fifty-nine (52%) patients developed an early severe graft dysfunction and needed an ECMO circulatory support within 24 h after HT. Data analysis revealed that among those patients, 47 (42%) had a severe primary graft dysfunction and 12 (11%) a secondary graft dysfunction due to acute rejection, hemorrhagic shock or pericardial tamponade. Forty-seven (80%) patients with ECMO were weaned from the circulatory support, while 12 patients (20%) died under support.

### Outcomes within 180 days after HT

The median ICU length of stay after HT was 16 [11–23] days, and the median hospital length of stay after HT was 34 [25–46] days. Ninety-four (83%) patients were alive 180 days after HT, and 8 out to 19 (42%) deaths were deemed to be caused by an infectious complication (pneumonia, *n* = 6, and intra-abdominal infection, *n* = 2). Bacterial infections in the ICU were associated with a longer stay in the ICU (20 days [95% CI 13–29] versus 12 days [95% CI 8–17] *p* < 0.001), but not associated with mortality. In an adjusted Cox model, the third non-viral infection was significantly associated with death at 90 days after HT (adjusted HR 6.2 [95% CI 1.2–31], *p* = 0.02).

## Discussion

We conducted a large single-center retrospective study to describe the epidemiology, risk factors and outcomes of infections occurring within 180 days after HT, in the era of high-priority allocations and mechanical support by ECMO and VAD. To our knowledge, it is the largest single-center cohort of recent adult heart transplant recipients analyzed for infections and their impact on prognosis. We found a very high rate of infections within 180 days after HT (*n* = 92, 81%), mostly bacterial or fungal infections (*n* = 80, 71%). Moreover, we identified four factors associated with non-viral infections depending on the postoperative period: hemodynamic instability at the time of HT, prior cardiac surgery, recipient age over 60 years and early circulatory support by ECMO following HT. According to the literature, the cumulative incidence of infections following HT varies from 30 to 80%, depending on the definitions of infections and the chosen interval time after HT [7, 10, 23, 24]. However, these studies have included different populations of recipients, as ECMO and VAD as a bridge to transplantation and national high-priority allocations were not as developed. Recently, Héquet et al. have showed in a national cohort study that 43% of the heart transplant recipients developed at least one bacterial or *Candida* infection within 1 year after HT. Interestingly the population in this study was different from ours as a higher proportion of the patients had a VAD before transplantation (18% vs 30%) [11]. In a recent cohort of pediatric heart transplant recipients, the authors demonstrated that 80% of the population had at least one infection following HT, but the mean period of follow-up was 5.3 years. In the same study, the instantaneous risk of acquisition of the first bacterial infection was the highest within 30 days following HT [25].

The most common bacterial and fungal infections in our study were pneumonia (52%) followed by skin and soft tissue infections (14%) in agreement with the literature [7, 10, 24]. The microbiological spectrum of infections did not differ from previous studies, Gram-negative bacilli being responsible for the majority of bacterial infections [10, 26, 27]. Our study also confirmed the increased incidence of MDR bacteria responsible for both carriage and infections before and after HT [28].

We showed that recipient age over 60 years, prior cardiac surgery (VAD excluded) and ECMO within 24 h after HT were risk factors associated with non-viral infections within 30 and 180 days after HT. Interestingly, VAD before HT and national high-priority allocation were not associated with infections following HT. National high-priority allocation criteria depend on the countries, but are everywhere designated for candidates on the waiting list who have the highest priority on the basis of medical urgency. However, in our study, hemodynamic instability at the time of HT, in the context of national high-priority allocation or not, was an independent risk factor for bacterial infection within 8 days after HT. Besides, recent papers showed that VAD before HT did not increase the risk of post-transplant infection [5, 11]. ECMO within 24 h after HT is considered as a severe graft failure, according to the ISHLT definition [21]. In our cohort, the incidence of ECMO support following HT was very high (52%). The criteria for a severe primary graft failure were found in 47 patients (42%). This incidence is higher than the one described in the literature (20% to 30%) [29, 30]. In France, the last national annual report recorded a recent increase in primary graft failure, with an incidence around 38% [31]. Age of both the donor and the recipient, ventilator dependence and ECMO at the time of HT, allosensitization before HT and cold ischemia time are known to be risk factors for primary graft failure [21, 29, 30, 32, 33]. Yet, those characteristics were not significantly associated with the occurrence of infections in our cohort of heart transplant recipients. Thus, ECMO following HT as a risk factor for early non-viral infection could be directly due to the external mechanical support but also to the recipient’s underlying condition, the donor’s characteristics and the surgery. Finally, prior cardiac surgery was already known as a potential risk factor for mortality from bacterial infection but not directly as a risk factor for bacterial infection [25].

Specific risk factors for invasive fungal infections would have been interesting to look for, as ECMO has already been described as an important risk factor in a HT population [34]. As we described only sixteen invasive fungal infections in our cohort, it would have been difficult to draw any conclusion on potential risk factors.

CMV infection was the predominant viral infection, occurring in 34% of the population. This rate is in accordance with recent studies focused on CMV [13, 14, 35]. However, the retrospective design of our study may represent an important source of bias for identification of symptomatic CMV disease, as symptoms such as diarrhea or isolated fever may have been missed. This high rate of CMV infections should be a prior concern, as it has been recently proved that CMV infection without any criteria of CMV disease could also be responsible for a reduction in 10-year cardiac allograft vasculopathy-free survival in heart transplant recipients [13, 35].

There was no difference in immunosuppressive regimen between patients developing or not infections after HT. However, the high rate of infections in our cohort was also a direct consequence of immunosuppressive therapies. In the current era of HT, those results enlighten the need of personalized immunosuppression, adjusted on patient’s risk factors for acute rejection and early infections [36, 37].

The overall mortality at 6 months after HT (17%) was in accordance with published data [4, 5, 33, 38]. Bacterial infections in the ICU were associated with an increased length of stay but not with mortality. In the literature, infectious complications after HT have been responsible for increased length of stay and healthcare costs in adults but also with increased mortality in children [7, 11, 25].

The first strength of this study was the inclusion of consecutive heart transplant recipients in a transplantation specialized center, in the era of VAD and ECMO mechanical supports and of increased rate of high-priority allocations. The detailed description of epidemiology, risk factors and outcomes of infectious complications in this new population could be a good tool for clinicians taking care of adult heart transplant recipients. We also acknowledge that the present study has several limitations. First, our peculiar population does probably not reflect the reality of all cardiac transplant centers, as the majority of enrolled patients were national high-priority allocations already hospitalized in ICU at the time of HT. It might be difficult to extend these results to all the patients on heart transplant waiting list. Moreover, the sample size is limited, yet it is a recent single-center study. We did not study the donor-derived infections even though it would have been of interest to better characterize early bacterial infections following heart transplantation. As the data collection was retrospective, potential inaccuracies in data reporting could exist. A few infectious episodes were also missing data, most notably the bacterial etiology of infection. Finally, strategies for perioperative management of heart transplant recipients may differ among institutions and may be considered a source of bias.

## Conclusion

This study confirmed the high rate of infections occurring within 180 days after HT in adult recipients. Recipient age, prior cardiac surgery, hemodynamic instability at the time of HT and ECMO following HT were independent risk factors for early and late non-viral infections. Thus, the recent pre- and post-transplant recipient characteristics modification could be directly correlated with infections following HT. A prospective study could be of interest to better determine risk factors for infections following HT.

## Additional file

**Additional file 1: Table S1.** Pre-, intra- and postoperative characteristics of heart transplant recipients without non-viral infection and patients developing at least one bacterial or fungal infection within 8 days, 30 days and 180 days after HT

## Abbreviations

95% CI: 95% confidence interval
BSI: bloodstream infection
CMV: cytomegalovirus
ECMO: extracorporeal membrane oxygenation
ESBL: extended spectrum beta lactamase
HT: heart transplantation
ICU: intensive care unit
ISHLT: International Society of Heart and Lung Transplantation
VAD: ventricular assist device
MDR: multi-drug-resistant
sHR: subdistribution hazard ratio
