# Supplementary material for: Infectious complications following heart transplantation in the era of high-priority allocation and extracorporeal membrane oxygenation
Source: Ann Intensive Care. 2019 Jan 25;9:17. doi: 10.1186/s13613-019-0490-2 (PMC6347647; doi:10.1186/s13613-019-0490-2)
Supplement: Supplementary file 1 — Additional file 1: Table S1. Pre-, intra- and postoperative characteristics of heart transplant recipients without non-viral infection and patients developing at least one bacterial or fungal infection within 8 days, 30 days and 180 days after HT [file 13613_2019_490_MOESM1_ESM.docx]

**Additional file 1: Table S1: Pre, per and post-operative characteristics of heart transplant recipients without non-viral infection and those developing at least one bacterial or fungal infection within 30 days and 180 days after HT**

|  | **Within eight days after HT** | | | **Within 30 days after HT** | | | **Within 180 days after HT** | | |
| --- | --- | --- | --- | --- | --- | --- | --- | --- | --- |
| **Characteristics** | **No bacterial infection (n=58)** | **≥ 1 bacterial infection(s) (n=55)** | **p value*** | **No bacterial or**  **fungal infection**  **(n=37)** | **≥ 1 bacterial or fungal infection(s) (n=76)** | **p**  **value**** | **No bacterial or**  **fungal infection**  **(n=33)** | **≥ 1 bacterial or fungal infection(s)**  **(n=80)** | **p**  **value***** |
| Recipient gender, male;  n (%) | 41 (71) | 45 (82) | 0.17 | 25 (68) | 61 (80) | 0.11 | 23 (70) | 63 (79) | 0.16 |
| Age, years;  median [IQR] | **50 [38 ; 58]** | **55 [43 ; 64]** | **0.08** | **47 [35 ; 55]** | **55 [44 ; 64]** | **0.01** | **47 [38 ; 56]** | **55 [43 ; 64]** | **0.03** |
| Prior cardiac surgery (VAD excluded); n (%) | **9 (16)** | **18 (33)** | **0.01** | **4 (11)** | **23 (30)** | **<0.01** | **4 (12)** | **23 (29)** | **0.01** |
| ICU at the time of HT;  n (%) | 21 (36) | 23 (42) | 0.61 | 14 (38) | 30 (40) | 0.94 | 14 (42) | 30 (38) | 1.0 |
| SOFA score at time of HT; median [IQR] | 3 [1 ; 6] | 4 [1 ; 6] | 0.42 | 3 [1 ; 6] | 4 [1 ; 6] | 0.71 | 4 [1 ; 6] | 4 [1 ; 6] | 0.69 |
| VAD before HT; n (%) | 9 (16) | 11 (20) | 0.57 | 4 (11) | 16 (21) | 0.23 | 4 (12) | 16 (20) | 0.34 |
| Mechanical ventilation before HT; n (%) | 12 (21) | 16 (29) | 0.21 | 8 (22) | 20 (26) | 0.37 | 7 (21) | 21 (26) | 0.36 |
| Epinephrine or Norepinephrine before HT; n (%) | **7 (12)** | **12 (22)** | **0.10** | 4 (11) | 15 (20) | 0.11 | **3 (9)** | **16 (21)** | **0.06** |
| ECMO before HT;  n (%) | 12 (21) | 16 (29) | 0.20 | 9 (24) | 19 (25) | 0.56 | 8 (24) | 20 (25) | 0.56 |
| Antibiotic treatment for bacterial infection at time of HT; n (%) | **15 (26)** | **7 (13)** | **0.09** | 9 (24) | 13 (17) | 0.21 | 8 (24) | 14 (18) | 0.23 |
| High priority HT; n (%) | 28 (48) | 30 (55) | 0.34 | 20 (54) | 38 (50) | 0.87 | 18 (55) | 40(50) | 0.88 |
| Cold ischemia, minutes; median [IQR] | 191  [149 ; 218] | 180  [127 ; 206] | 0.22 | 189  [129 ; 210] | 189  [140 ; 210] | 0.64 | 190  [156 ; 210] | 187  [127 ; 210] | 0.44 |
| Packed red blood cells transfused;  median [IQR] | 2 [0 ; 4] | 3 [0 ; 5] | 0.31 | 2 [0 ; 4] | 3 [0 ; 5] | 0.23 | 2 [0 ; 4] | 3 [0 ; 5] | 0.31 |
| Donor gender, male;  n (%) | 30 (52) | 37 (67) | 0.10 | 21 (57) | 46 (61) | 0.43 | 18 (55) | 49 (61) | 0.35 |
| Donor age, years; median [IQR] | 46 [37 ; 52] | 44 [37 ; 53] | 0.83 | 47 [38 ; 52] | 44 [37 ; 52] | 0.42 | 47 [40 ; 52] | 44 [36 ; 52] | 0.33 |
| Thymoglobulin induction; n (%) | 53 (92) | 48 (87) | 0.26 | 33 (89) | 68 (90) | 0.43 | 30 (91) | 71 (89) | 0.22 |
| ECMO after HT; n (%) | **23 (40)** | **36 (66)** | **0.02** | **13 (35)** | **46 (61)** | **0.01** | **12 (36)** | **47 (59)** | **0.02** |
| SOFA score day 1 after HT; median [IQR] | **8 [7 ; 10]** | **10 [8 ; 11]** | **<0.01** | **8 [7 ; 10]** | **10 [8 ; 11]** | **<0.01** | **8 [7 ; 9]** | **10 [8 ; 11]** | **<0.01** |
| Plasma exchange after HT; n (%) | 11 (19) | 9 (16) | 0.59 | 7 (19) | 13 (17) | 0.53 | 7 (21) | 13 (16) | 0.40 |

ECMO: extracorporeal membrane oxygenation; HT: heart transplantation; ICU: intensive care unit; SOFA score: sequential organ failure assessment score; VAD: ventricular assist device

* p value comparing patients with no bacterial infection and those with at least one bacterial infection within eight days after HT

** p value comparing patients with no bacterial or fungal infection and those with at least one non-viral infection within 30 days after HT

*** p value comparing patients with no bacterial or fungal infection and those with at least one non-viral infection within 180 days after HT
